# Gender-specific predictors of poor sleep quality and insomnia among caregivers of persons living with dementia

**DOI:** 10.1093/geroni/igab046.2983

**Published:** 2021-12-17

**Authors:** Glenna Brewster, Miranda McPhillips, Kate Yeager, Fayron Epps, Kalisha Bonds Johnson, Kenneth Hepburn, Donald Bliwise

**Affiliations:** 1 Emory University, Stonecrest, Georgia, United States; 2 University of Pennsylvania, University of Pennsylvania, Pennsylvania, United States; 3 Emory University, Atlanta, Georgia, United States

## Abstract

Caregiving for a person living with dementia (PLwD) may influence sleep patterns. Gaps exists about whether caregiver and PLwD factors impact sleep differentially based on caregivers’ gender. The aim of this secondary data analysis was to identify predictors of sleep quality and insomnia in a sample of caregivers, stratified by gender, participating in a randomized controlled trial of a psychoeducational intervention. Outcome measures were sleep quality (Pittsburgh Sleep Quality Index, PSQI) and insomnia (Insomnia Severity Index, ISI). Participants (n=261) also completed measures about caregivers’ perceived stress, burden, depression, and self-care, and PLwD’s behaviors (i.e., apathy, sleep disorders). Linear regression modeling was used to identify the overall predictors of poor sleep quality (PSQI > 5; 52% of the sample) and insomnia (ISI > 7; 41% of the sample). Caregivers were primarily female (70.5%), White (73.6%), mean age of 64.6 (±11.2) years, and typically caring for a spouse (65.9%). For male caregivers, predictors of poor sleep quality were assisting the PLwD with instrumental activities of daily living and PLwD neuropsychiatric symptoms (F=4.45, p<.001); while caregiver self-care and PLwD neuropsychiatric symptoms predicted insomnia (F=4.49, p<.001). For female caregivers, the predictors of poor sleep quality were caregiver depressive symptoms and burden, and frequency of PLwD behavioral problems (F=4.46, p<.001); however, only perceived stress predicted insomnia (F=4.32, p<.001). Various factors related to caregiving appear to be more important than others in predicting sleep outcomes of male/female caregivers. Health care professionals should acknowledge gender differences when designing and implementing programs and interventions to improve sleep.

